# Genome Sequence of Saccharomyces cerevisiae NCIM3107, Used in Bioethanol Production

**DOI:** 10.1128/genomeA.01557-14

**Published:** 2015-02-12

**Authors:** Kandasamy Ulaganathan, Burragoni Sravanthi Goud, Mettu Madhavi Reddy, Vanaparthi Praveen Kumar, Surabhi Radhakrishna, Jatoth Balsingh

**Affiliations:** Centre for Plant Molecular Biology, Osmania University, Hyderabad, Telangana, India

## Abstract

Here, we report the genome of Saccharomyces cerevisiae strain NCIM3107, used in bioethanol production. The genome size is approximately 11.8 Mb and contains 5,435 protein-coding genes.

## GENOME ANNOUNCEMENT

An ideal organism for lignocellulosic bioethanol production should be able to utilize lignocellulose, ferment hexose and pentose sugars, have a high ethanol yield, and tolerate high ethanol concentration (1, 2). Although a number of organisms, like Escherichia coli, Zymomonas mobilis, Clostridium species, and Trichoderma reesei, have been tried for use in lignocellulosic ethanol production, Saccharomyces cerevisiae is the most widely used organism (3, 4). S. cerevisiae meets most needs for a bioethanol producer, and its inability to utilize pentose sugars has been addressed by genetic engineering (4–6). Understanding the genetic background of the strain is important for its use in industrial production and metabolic engineering. As part of our effort to select a suitable host strain for the heterologous expression of genes associated with lignocellulosic bioethanol production, we sequenced the NCIM3107 strain of S. cerevisiae obtained from the Microbial Type Culture Collection, Chandigarh, India. This strain has been tested for bioethanol production and found to be a moderate producer compared to other tested strains (7). Recent comparative genomics work has revealed that many yeast strains possess unique variations and genes, so sequencing the genome would be the first step in understanding the genetic background of a strain to be used in industrial production and for use in metabolic engineering (8–12). Here, we report the genome sequence of S. cerevisiae strain NCIM3107.

The genome was sequenced with the Illumina MiSeq system. A total of 1,459,146 reads were generated, with a coverage of 27.35-fold. The genome was assembled using the Genotypic tool. A genome of 11.8 Mb was assembled, covering 16 chromosomes. The raw reads obtained were aligned to the reference S. cerevisiae R64 genome with the Bowtie 2 tool (13). Using SAMtools, the variants were detected in comparison to the S. cerevisiae R64 reference genome, with cutoffs of ≥20-read depth and ≥30 mapping quality score (14). The variants detected with SAMtools were further annotated using SnpEFF 3.4 to give the locations (intronic/exonic/untranslated region [UTR]), gene names, protein changes, and functions of the variants (15).

Gene prediction and annotation were performed using the Augustus software with the training sets available for S. cerevisiae (16). There were 5,435 protein-coding genes, which showed homology to S. cerevisiae genes found in the Saccharomyces Genome Database (SGD) (17).

### Nucleotide sequence accession numbers.

The nucleotide sequences of the S. cerevisiae NCIM3107 genome have been deposited in GenBank under the accession numbers CP009944 to CP009960.
